# Orangutan mothers adjust their behaviour during food solicitations in a way that likely facilitates feeding skill acquisition in their offspring

**DOI:** 10.1038/s41598-021-02901-z

**Published:** 2021-12-08

**Authors:** Mulati Mikeliban, Belinda Kunz, Tri Rahmaeti, Natalie Uomini, Caroline Schuppli

**Affiliations:** 1 Development and Evolution of Cognition Research Group, Max Planck Institute of Animal Behavior, 78467 Konstanz, Germany; 2 Department of Anthropology, University of Zurich, CH-8006 Zurich, Switzerland; 3 Department of Biology, Graduate School, Universitas Nasional, Jakarta, 12520 Indonesia; 4 Department of Linguistic and Cultural Evolution, Max Planck Institute for Evolutionary Anthropology, 04103 Leipzig, Germany

**Keywords:** Evolution, Anthropology, Biological anthropology

## Abstract

Immature orangutans acquire their feeding skills over several years, via social and independent learning. So far, it has remained uninvestigated to what extent orangutan mothers are actively involved in this learning process. From a fitness point of view, it may be adaptive for mothers to facilitate their offspring’s skill acquisition to make them reach nutritional independence faster. Food solicitations are potential means to social learning which, because of their interactive nature, allow to investigate the degree of active involvement of the mother. To investigate the role of food solicitation and the role of the mother in immatures’ foraging skill acquisition, we analysed 1390 food solicitation events between 21 immature Sumatran orangutans (*Pongo abelii*) and their mothers, collected over 13 years at the Suaq Balimbing orangutan population. We found that solicitation rates decreased with increasing age of the immatures and increased with increasing processing complexity of the food item. Mothers were more likely to share complex items and showed the highest likelihoods of sharing around the age at which immatures are learning most of their feeding skills. Our results indicate that immature Sumatran orangutans use food solicitation to acquire feeding skills. Furthermore, mothers flexibly adjust their behaviour in a way that likely facilitates their offspring’s skill acquisition. We conclude that orangutan mothers have a more active role in the skill acquisition of their offspring than previously thought.

## Introduction

With weaning at the age of 6.5–9 years, orangutans (*Pongo *spp.) show one of the longest periods of nutritional dependence in mammals^1^. Orangutans mothers get pregnant only after they have weaned their previous offspring, which leads to very long interbirth intervals and thus slow reproductive rates^1^. The long nutritional dependence is likely a result of a combination of factors, including the orangutan’s semi-solitary life-style (solitary life-style hypothesis^2,3^): immature orangutans start ranging independently from their mothers shortly after weaning^2,3^ and from that point onward spend between 60 and 90 percent of their time on their own^3^. Hence, because they cannot resort to the knowledge of association partners, to be able to be weaned immature orangutans must have their skills sufficiently in place before leaving their mother^2,3^.

Immature orangutans must acquire a broad range of ecological skills to become self-sufficient, which includes learning to locate, identify and process^4^ more than 200 different food items (i.e., the combination of the species and the part of the species that is eaten)^5^. Most of these food items require a distinctive combination of manual and oral processing steps before they can be ingested^6,7^ with some items even requiring the use of stick tools for their extraction^8^. Previous studies showed that it takes immature orangutans around 8 years to establish the full extent of their diet repertoires^6^. Between the ages of 8 and 12 years, adult-level food processing skills are reached while competence is only reached later for complex to process (i.e., items that require more steps of pre-ingestive processing) compared to easy to process food items (i.e., items that require fewer steps of pre-ingestive processing)^6,7^.

Previous studies showed that immature orangutans rely on social and independent learning mechanisms to acquire their feeding skills. In terms of social learning, observational social learning through peering (i.e., sustained close range watching of a conspecific's behaviour^7^) appears to play a major role. Immature Sumatran and Bornean orangutans (*Pongo abelii* and *Pongo pygmaeus wurmbii*) show selective practice of the observed behaviour after peering^7^. They also peer more frequently at items that are more complex to process and/or rarer in the population's diet (i.e., items for which they receive fewer learning opportunities^7^). In other words, immature orangutans peer most frequently at items that are more difficult to learn.

In addition to peering, soliciting food (i.e., attempts by an individual to obtain a food item from another individual by using gestures, which is commonly also referred to as begging) may be a way to socially acquire knowledge for immature orangutans^9,10^, as it is in other species, including primates^11–20^ and non-primates^21–31^. Bornean immature orangutans solicit food items that are more complex more frequently than easier to process ones^10^. Furthermore, as they get older and more competent, Bornean immature orangutans solicit food less frequently and solicit a smaller variety of food items^10^. These findings suggest that soliciting food is a way through which immature Bornean orangutans acquire information about how to identify and process food items, as predicted by the informational hypothesis^32^.

In both of these orangutan social learning pathways (i.e., learning through peering^7^ and learning through soliciting food^9,10^), the orangutan mother serves as the primary role model^3^. These findings are in line with the results from a broad range of species (including all non-human great apes) which suggest that learning from their primary caregiver is the main social learning strategy during early immaturity (reviewed in^33^). In contrast to social learning through peering, during learning through food solicitations and subsequent food transfers, the role model plays a potentially more active role. In many species, including orangutans, the degree of active involvement of the role model during food solicitations remains yet to be investigated. In general, the nature of food transfer events can range from being requested by the offspring to being proactively initiated by the role model^24,34–36^. Food transfers can be instances of teaching. Teaching is functionally defined as when an actor (1) only alters its behaviour in the presence of a naive observer, (2) the behavioural change comes at a cost or without any immediate benefit for the actor, and (3) the behavioural change of the actor ultimately leads to the acquisition of new skills or knowledge or to an increase in learning speed in the observer^37^. Thus, if a food transfer satisfies these criteria, it can be considered an instance of teaching.

A series of studies on food transfers showed that beyond serving as passive role models, in chimpanzees (*Pan troglodytes*), mothers have an active role in the skill acquisition of their offspring^38–41^. Chimpanzee mothers scaffold (sensu^40^) learning opportunities during extractive foraging with tools by sharing their tools with their offspring^40,41^. Obtaining their mother's tool increases the performance of the offspring and in some cases, decreases the mother’s efficiency^40,41^. Furthermore, across chimpanzee study sites, tool transfers vary with task complexity^42^. Accordingly, tool sharing in chimpanzees may qualify as functional teaching^41^.

Beyond primates, food sharing in other taxa plays an essential role for offspring to learn foraging skills. For example, in some other species, the mother actively gives food to her pup and the pup also takes food from her; through these interactions sea otter pups (*Enhydra lutris*) likely acquire a tool-use preference and diet specialization^43–46^). In New Caledonian crows (*Corvus moneduloides*), while using tools to obtain food, adults feed juveniles, tolerate close physical contact, and allow them to steal their food and tools; the young crows thus likely learn to produce a tool type and how to use it^47–49^. Thus, for species that use tools, at least, there is a clear benefit of social learning through interactions with adults during foraging. However, to date it has remained uninvestigated if orangutan mothers have such an active role in this learning process as opposed to serving as passive role models.

Orangutans are particularly interesting and well suited to investigate the mother's involvement in skill acquisition through food transfers because of their long offspring dependency (i.e., lengthy interbirth intervals^1^), semi-solitary lifestyle^50^, and complex foraging niche^51^. Because of this combination, from a fitness point of view, it may be adaptive for orangutan mothers to support their offspring's skill acquisition because increasing the speed at which their offspring reach nutritional independence will likely speed up the mothers’ reproduction^52^. During the dependency period (i.e., 0–8 years^1^), immature orangutans frequently solicit food from their mothers^3,10^. The mothers’ behavioural responses during these events vary in their level of tolerance, ranging from tolerant, which is letting the offspring take the food item, to intolerant, such as refusing to let the offspring take the food item by holding onto the food item or by showing low-level aggression towards the offspring^10^. This behavioural plasticity may be a way through which orangutan mothers flexibly facilitate skill learning in their offspring and it calls for investigating the possible presence of teaching.

In this study we investigate food solicitations and subsequent food transfers as potential means to social learning in a population of Sumatran orangutans at Suaq Balimbing, Indonesia. In particular, we examine the degree of active involvement of the role model in this potential means to social learning. The Suaq Balimbing habitat shows higher levels of food availability than most other orangutan habitats which goes in hand with reduced feeding competition^53^. The orangutans at Suaq Balimbing also show broader and more processing-intense diet repertoires (including the regular use of stick tools in different contexts) than other orangutan populations^54–56^. Both the high social tolerance and complex diet repertoires make the Suaq Balimbing orangutans especially suitable to investigate social learning through food solicitations.

Whereas for Bornean orangutan immatures a previous study found evidence that food solicitations are used as a means to learn about food processing^10^, this link has never been established for Sumatran orangutans. Therefore, in a first step we investigate if food solicitations are also used as means to social learning in Sumatran orangutans. Due to the considerably higher data availability including a larger number of individuals, broader offspring age range and denser data, we can examine patterns in more detail than the Bornean study^10^. In a second step, we investigate to what degree orangutan mothers are actively involved in the food solicitations as opposed to serving as a passive role model only. We do this by investigating whether orangutan mothers adjust their behaviour during food solicitation events in a way that facilitates the acquisition of everyday feeding skills in their offspring. We discuss our findings in comparison to results on skill acquisition in chimpanzees and humans and to what extent they are in line with teaching.

### Predictions

1. We predicted that Sumatran orangutans use food solicitations to learn how to identify and process food items. In this context, we tested if immature orangutans solicit food less frequently with increasing age (i.e., increasing their own ecological competence), and solicit food more frequently with increasing pre-ingestive processing complexity of the food item and decreasing frequency at which they encounter the food item. Because previous studies have shown that immatures reach competence with complex items later^6^, we also tested for an interaction of the effects of age and food item processing complexity on food solicitations.
2. If Sumatran orangutan mothers facilitate skill development in their offspring through flexible behavioural adjustments during food solicitations, we predicted the mothers would show decreasing levels of tolerance with increasing age (i.e., increasing ecological competence levels) of the offspring. We also predicted that they would show increasing levels of tolerance with increasing processing complexity and decreasing levels of tolerance with increasing frequency of the food item in the food repertoire of population. Furthermore, because immatures reach processing competence for complex food items later than for easy to process ones^6^, we predicted an interaction between the effects of age of the offspring and the complexity of the food item on the level of tolerance of orangutan mothers to their offspring’s food solicitation behaviour.

## Methods

### Study site and study period

The data for this study were collected on a population of Sumatran orangutans at the Suaq Balimbing research station (3°42′N, 97°26′E) in the Gunung Leuser National Park, South Aceh, Indonesia by trained students, researchers, and field assistants from June 2007 until January 2020 (data were collected in 131 months).

### Data collection

Data were collected during focal animal follows wherein the activity of the focal animal was assessed through scans at 2-min intervals, using standardized paper data sheets or their electronic versions on tablets (iPad mini 4). In case the activity of the focal animal was feeding, the food species, as well as the part that was ingested, was recorded on the data sheets. For plant parts, we differentiated between leaves, fruits, flowers, bark, pith, or other vegetative matter. For insect parts, we differentiated between the insect itself (e.g., ants, termites, bees, sweat bees) and its different products (e.g., honey or eggs). Additional food items included eggs, meat of small vertebrates and water. We refer to each specific combination of a species and part as a “food item”.

Food solicitation (i.e., begging) was defined as attempts by an individual to obtain a food item from another individual by reaching towards the food with its hand or mouth (with or without actually touching the food) or by directly grabbing the food item from the hands or mouth of another individual. The recording of food solicitation events happened at an all-occurrence basis, using data sheets on paper or on tablets and on all individuals present during the focal follow. Between two consecutive food solicitation events, for them to be scored as two separate events, there had to be a pause of at least 10 s where the focal individual was engaging in a different activity than soliciting food. For each food solicitation event, the identity of the soliciting individual, the identity of the target individual, the solicited food item, and the outcome of the solicitation event (i.e., if the solicitor obtained the food or not) were noted.

### Focal animals, datasets and variables

We had focal data on 27 immatures (including 10 females and 17 males) and their mothers (N = 13; see Supplementary Table 1 for an overview of the focal individuals) available. All these individuals are part of the long-term data collection at Suaq. These data included a total of 4787 observation hours (collected during 475 focal follows) on the immatures and 4220 observation hours (collected during 435 focal follows) on their mothers.

In total, 1390 food solicitation events by 21 immatures between the age of 0.3 and 13.2 years (M_age in years_ = 3.7) were recorded. 1371 food solicitations (i.e., 98.6 percent) were directed at the mother. Because on most days, mothers and their offspring (i.e., immature) were followed simultaneously, for the food solicitation events directed at the mother, we were able to control for the opportunities the immatures had to solicit these items on each day with simultaneous data through the mother's feeding record on that day.

We analysed the effects of different independent variables on three dependent variables:

(I) Food solicitation frequencies, as an overall approximation of how often immature orangutans solicit food. Food solicitation frequencies were calculated daily (i.e., per focal follow) and by dividing the total number of food solicitation events (directed at all classes of target individuals) by the total number hours the focal individual was observed on that day. To calculate food solicitation frequencies (*dataset 1*), we only used full day follows and only follows and food solicitation events in which the food solicitor was the focal individual (i.e., the individual for whom the focal behavioural data were collected) or the offspring of the focal individual (for 95 days on which only data on the mother were available). Because food solicitations are inherently dependent on the opportunities immatures have to solicit a specific food item, we also calculated food solicitation rates.
(II) Food solicitation rates represent the relative daily frequency at which immatures solicit a specific food item from their mothers, controlled for the opportunities they have to solicit that same food item on that day. We assessed these opportunities by counting the number of 2 min scans at which its mother fed on each food item on a given day^57^. In our statistical models we implemented this with a Poisson approach (see below). For the analyses on food solicitation rates, we only included follows of which the food solicitor was the focal individual and for which also simultaneous focal behavioural data on the mother were available (*dataset 2*).
(III) As a measure of the tolerance of the orangutan mother during a food solicitation event, we looked at its outcome, using a binary coding system. An event was classified as successful if the focal individual managed to retrieve the food item from their mother and as unsuccessful if the focal did not manage to obtain the food item (*dataset 3*). For the analyses on tolerance, we included all available food solicitation events.

In terms of the independent variables, we included the age of the offspring, as well as the complexity, and frequency of the food item. Complexity was assessed via the number of manual and oral steps it took to process a food item before ingestion and ranged from 0 to 5. Complexity 0 referred to simple pluck and eat (e.g., eating leaves) and each additional step in between such as peeling, biting in half, spitting out seeds counted as an extra step (1–4). Processing complexity 5 refers to tool use (see^7^ for more details and examples). The frequency of the food item was approximated by the abundance of the food item in the diet of the Suaq Balimbing resident population, which was calculated by its relative occurrence in all the 2-min feeding scans collected on the resident adult females at Suaq Balimbing over the 12-year study period (N = 136,161).

### Statistical analyses

All statistical analyses and plots were done in the R programming language^58^. To test our predictions, we used three statistical models, one for each of the three dependent variables and datasets described above.

With *model 1* (i.e., daily food solicitation frequency model) we looked at the effects of offspring age on the overall daily food solicitation frequency using *dataset 1*. The dataset for this analysis consisted of 416 daily food solicitation frequencies (N_zero frequencies_ = 115, N_non-zero frequencies_ = 301). We used a generalized linear mixed model (GLMM^59^) with Gaussian family distribution, the glmmTMB function from the glmmTMB package^60^, and the bbmle package for the maximum likelihood estimation (REML = F^61^). For this model, we included two random intercepts: the offspring (N = 22) and the observer (i.e., the main observer of the focal follow, N = 34). The intercept of the offspring controls for potential systematic differences between individuals throughout their development. The intercept of the observer controls for potential observational differences. We also included the exact age of the offspring as a random slope within both random intercepts. To avoid elevating the rate of type I error^62^, we maximized the number of random slopes in our models.

With *model 2* (i.e., daily food solicitation rate model) we looked at effects of the offspring age, the complexity and frequency of the food item on the overall food solicitation rates, using *dataset 2*. The dataset for this analysis consisted of 3071 daily food solicitation rates (whereby N_daily food solicitation rates,_ = N_different food item and day combinations_ = 3071, N_daily food solicitation rates equaling zero_ = 2639, N_non-zero daily food solicitation rates_ = 432) for which we had available the information on all the variables of our model. Based on the results of previous studies^6^, we expected that the complexity of the food item may impact the relationship between the offspring’s age and their food solicitation rate. We therefore also included an interaction between the age variable and the complexity variable. Because the GLMM of the full model with a Poisson family distribution indicated overdispersion (dispersion parameter = 2.99) we used GLMMs with negative binomial family distribution (which showed no more overdispersion issues: dispersion parameter = 0.79), using the glmmTMB function from glmmTMB package^60^ for this analysis. Because all the relationships in this model were linear, we used the nbinom1 function from the glmmTMB package^60^. We also used the bbmle package for the maximum likelihood estimation^61^. For this model, we included three random intercepts in the model: the offspring (N = 20), the observer (N = 35), and the food item (N = 180). The intercept of the food item controls for potential systematic differences between the items. We also included three random slopes: the exact age of the offspring, as well as the complexity and the frequency of the food item.

With *model 3* (i.e., food solicitation success model), we analysed the effects of the age of the offspring, complexity of the food item, and frequency of the food item on the outcome (i.e., success) of a food solicitation event. Because previous findings suggest that the complexity of food items may impact the relationship between the offspring’s age and their competence level^6^, we also included an interaction between the age variable and the complexity variable. For this analysis we used *dataset 3*, which consisted of 918 out of 1381 total food solicitation events for which we had the information on all the variables of the model available. Because the dependent measure of this model is the tolerance of the orangutan mother during the food solicitation event, we excluded the food solicitation events where the target was not the mother (N = 18). Because the response variable is binary, we used a GLMM with binomial family distribution and logit link function^63^. We also used the glmmTMB function from the glmmTMB package^60^, and the bbmle package for the maximum likelihood estimation^61^. We included three random intercepts in the model: the offspring (N = 17), the observer (N = 34), and the food item (N = 92). We also included three random slopes: the exact age of the offspring, complexity of the food item, and frequency of the food item.

Before fitting each of the models, we used the psych package^64^ to look at each of the numeric dependent and independent variables (i.e., food solicitation frequency, food solicitation rate, age, complexity, and frequency) in detail (i.e., we examined the mean, standard deviation, minimum, maximum, median, and skewness and kurtosis of the variable). For the Gaussian model (i.e., model 1), the plotQQunif function from the DHARMa package^65^ indicated normally distributed residuals. Additionally, to rule out multicollinearity for all three models, we determined Variance Inflation Factors (VIF^66^) by using standard linear models which had the same structures as the original mixed models’ but did not include any random effects. None of the three models showed multicollinearity.

After fitting each of the three models, as a comprehensive test of the overall effect of the fixed effects, we compared the full model to a null model which lacked all fixed effects but included the same random effects structure as the full model^67^ using a likelihood ratio test^68^. For testing the effect of each individual variable, we dropped the variable from the model and compared the reduced model to the complete model via a likelihood ratio test.

Because previous studies had shown that in orangutans social learning frequencies increase during early immaturity and then decrease again during late immaturity (i.e., they have a negative quadratic relationship with age^7,69^), in each of the three models, we tested for a quadratic effect of age by comparing the original model which included the linear age variable to a model which included the age variable as both linear and quadratic, using a maximum likelihood test. In these model comparisons, we kept the same random effects (i.e., the observer and offspring intercepts and the slope of age within the offspring intercept) in all compared models.

Furthermore, based on the fact that immature male and female chimpanzees solicit food differently and that their mothers’ tolerance toward food solicitations differs depending on the sex of the offspring^40,70,71^, we investigated a potential effect of offspring sex in all our three final models by testing if the sex variable increased the goodness of model fit using likelihood ratio tests^68^. Our results revealed that the sex variable neither had an effect in any of our models nor did it increase the model fit of any of our models (see Supplementary Table 3 and Supplementary Figs. 1–3 for the raw data inspections). Hence, we did not keep the sex variable in any of our final models.

We used the ggplot2 package^72^ for creating the plots of the model predictions (i.e., the predicted probability of the outcome) and the plots of the raw data which we show in the appendix.

### Ethics declarations

Our research was approved by the Indonesian State Ministry for Research, Technology and Higher Education (RISTEKDIKTI; Research Permit No.: 152/SIP/FRP/SM/V/2012 and following) and adhered to the legal requirements of Indonesia. Our data collection was merely observational with an at least 7-m distance between the focal individual and the observer and was thus non-invasive. The observers did not interact with the study animals in any way and thus avoided influencing the natural behaviour of the individuals.

## Results

### Prediction 1

To investigate if immature Sumatran orangutans use food solicitations as means to acquire their feeding skills, we looked at the effect of the offspring's age on daily food solicitation frequency, using model 1 with *dataset 1.* We also looked at the effect of the offspring's age, as well as complexity and frequency of the food item on the daily food solicitation rate, using model 2 with *dataset 2*.

**Figure 1:**
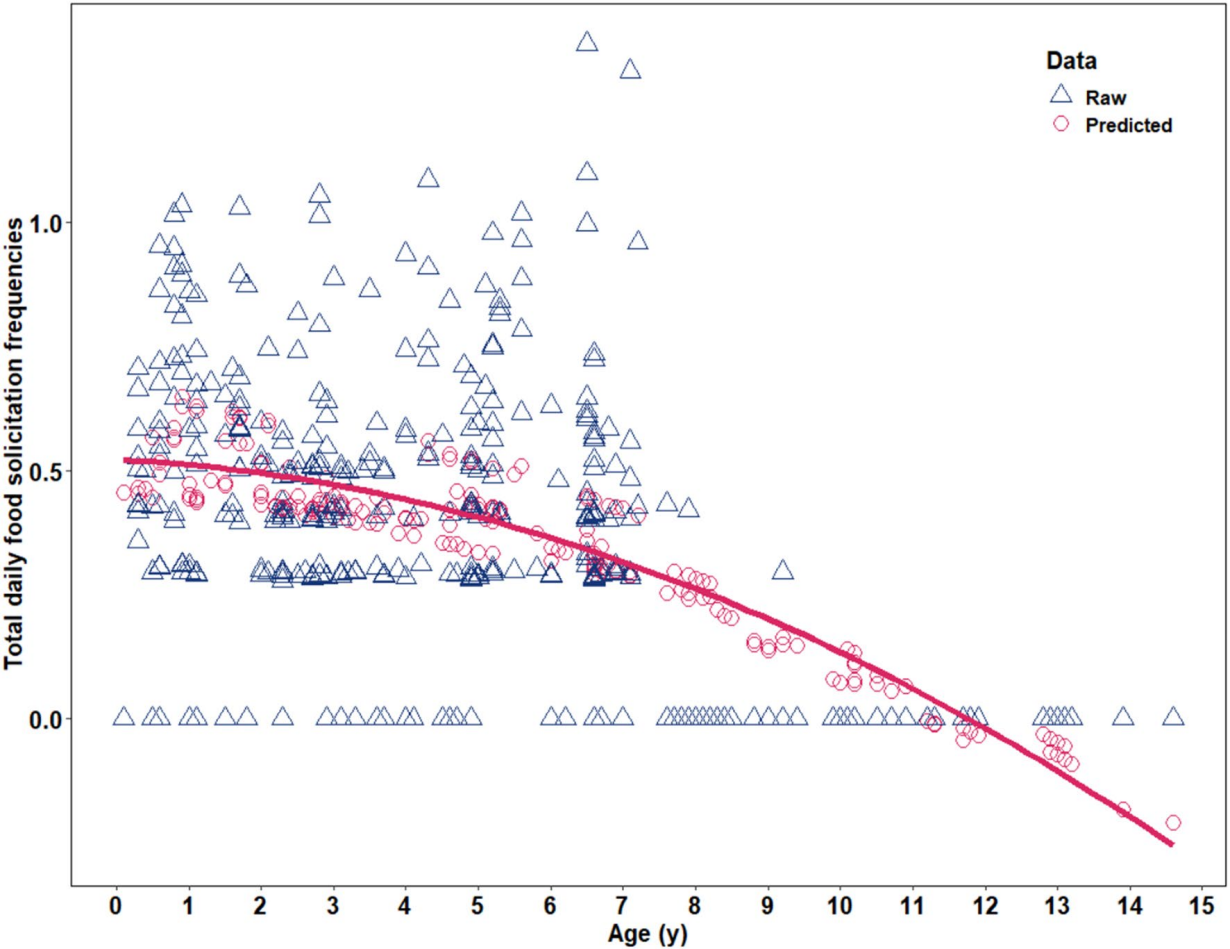
Effect of the offspring’s age (in years) on predicted food solicitation frequencies (square root transformed; model 1*, dataset 1*, N = 416 food solicitation frequencies). The blue triangles represent the raw daily food solicitation frequencies (events per hour). The pink circles are the predicted daily food solicitation frequencies. The pink curve shows the fitted probability at the 95% level.

For the food solicitation frequency model (model 1), when determining the nature of the age effect before fitting the final full model, the full model with age as a quadratic and a linear variable fitted the data better than the full model with age as a linear variable only (χ^2^ = 4.96, df = 1, p = 0.03, Supplementary Table 2). Therefore, we kept the age variable being both linear and quadratic in the full model. Furthermore, the full model fitted the data better than the null model which only included the random effects (likelihood ratio test: χ^2^ = 20.55, df = 2, p < 0.01). In terms of the individual effects, the final full model revealed a significant negative quadratic effect of age on food solicitation frequency (Table 1: χ^2^_linear age_ = 19.42, df_linear age_ = 1, p_linear age_ < 0.001, χ^2^_quadratic age_ = 4.96, df_quadratic age_ = 1, p_quadratic age_ = 0.03), which indicates that as the offspring gets older, the daily total food solicitation frequency decreases (Fig. 1).

**Table 1:** Effects on all three models. Estimates and standard errors (retrieved from the summary output of the GLMM with a binomial error distribution), chi-square (χ^2^), confidence intervals (95% CI) [lower, upper] degrees of freedom, p values (obtained via a likelihood ratio test by dropping each of the fixed effects one at a time and comparing the reduced model to the complete model) for all three models. Significant values are in bold.

Response variable	Estimate	SE	Lower CI	Upper CI	χ^2^	*df*	p
**Food solicitation frequency [model 1]**							
(Intercept)	0.39	0.03	0.33	0.44	a	a	a
Immature age^b^	− 0.13	0.02	− 0.16	− 0.10	19.42	1	**< 0.001**
(Immature age)^2b^	− 0.03	0.01	− 0.05	0	4.96	1	**0.026**
**Food solicitation rate [model 2]**							
(Intercept)	− 4.68	0.20	− 5.10	− 4.28	a	a	a
Immature age^c^	− 0.40	0.09	− 0.58	− 0.22	a	a	a
Frequency^d^	− 0.35	0.22	− 0.78	0.07	2.33	1	0.127
Complexity^e^	0.45	0.10	0.25	0.64	a	a	a
Immature age^c^: Complexity^e^	0.21	0.06	0.10	0.32	14.47	1	**< 0.001**
**Food solicitation success [model 3]**							
(Intercept)	0.26	0.25	− 0.22	0.75	a	a	a
Immature age^f^	0.36	0.13	0.09	0.62	a	a	a
(Immature age)^2f^	− 0.37	0.11	− 0.59	− 0.15	11.42	1	**0.001**
Frequency^g^	0.13	0.09	− 0.04	0.31	1.99	1	0.158
Complexity^h^	0.02	0.12	− 0.21	0.26	a	a	a
Immature age^f^: Complexity^h^	0.23	0.10	0.04	0.42	5.71	1	**0.017**

^a^Not shown, as having limited interpretation.

^b^z-transformed to a mean of zero and SD of one; mean and SD of the untransformed variable were 4.95 and 3.34 processing steps, respectively.

^c^z-transformed to a mean of zero and SD of one; mean and SD of the untransformed variable were 4.07 and 2.46 processing steps, respectively.

^d^z-transformed to a mean of zero and SD of one; mean and SD of the untransformed variable were 0.05 and 0.07 processing steps, respectively.

^e^z-transformed to a mean of zero and SD of one; mean and SD of the untransformed variable were 1.62 and 1.25 processing steps, respectively.

^f^z-transformed to a mean of zero and SD of one; mean and SD of the untransformed variable were 3.66 and 2.15 years, respectively.

^g^z-transformed to a mean of zero and SD of one; mean and SD of the untransformed variable were 0.05 and 0.06, respectively.

^h^z-transformed to a mean of zero and SD of one; mean and SD of the untransformed variable were 2.45 and 1.31 processing steps, respectively.

For the model on the food solicitation rates (model 2), before fitting the final full model, the full model with age as a linear factor and the full model that additionally included age as a quadratic variable showed no significant difference (χ^2^ = 0.92, df = 1, p = 0.34, Supplementary Table 2). Therefore, we kept the linear age variable in the model only. Furthermore, the full model, which included the variable linear offspring age, complexity of the food item, frequency of the food item, the interaction between the linear age variable and the complexity variable, and the random effects, fitted the data better than the null model which only included the random effects (likelihood ratio test: χ^2^ = 36.64, df = 4, p < 0.001), indicating an overall effect of the four variables on the response variable. In terms of the individual effects, the full model revealed a significant effect of the interaction between offspring age and the complexity of the food item on the food solicitation rate (Table 1: χ^2^ = 14.47, df = 1, p < 0.001).

**Figure 2:**
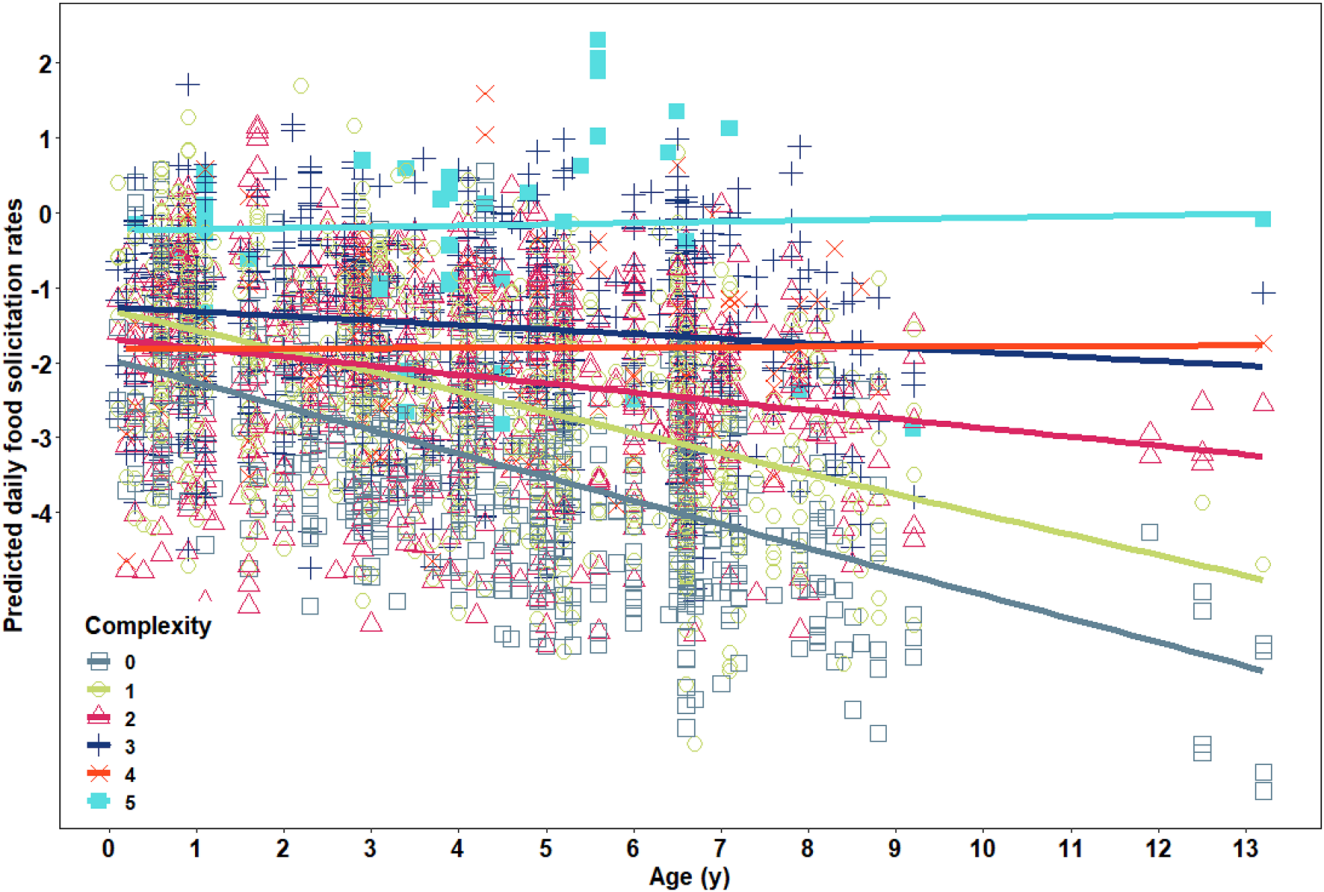
Effects of the offspring’s age (in years) and the complexity of the food item on predicted food solicitation rates. Complexity was assessed via the number of manual and oral steps it took to process a food item before ingestion and ranged from 0 to 5. The food solicitation opportunities (i.e., the number of feeding bouts of the mother) were log transformed. The lines show the fitted probability at the 95% level (model 2*, dataset 2*, N = 3071 food solicitation rates).

The nature of the interaction effect between age and food solicitation rate indicated a slowdown in the decrease of food solicitation rate over offspring age with increasing item processing complexity (Fig. 2, see Supplementary Fig. 2 for the raw data). In other words, the less complex foods showed a faster drop in solicitation, whereas the more complex foods had a higher solicitation rate from the start and dropped at a later age. The most complex food had the highest solicitation rates and stayed constant across age. The full model indicated no effect of the frequency of the food item on the food solicitation rate in Table 1.

### Prediction 2

To investigate the mother's role in their offspring's acquisition of feeding skill, we looked at the effect of the offspring's age, complexity of the food item, and frequency of the food item on the mother's tolerance to the food solicitation behaviour using model 3 with *dataset 3*.

Before fitting model 3, when comparing the full model with age as a linear factor only to the model that additionally included age as a quadratic variable, we found a significant difference (χ^2^ = 8.92, df = 1, p = 0.003, Supplementary Table 2). Therefore, we kept the age variable being both linear and quadratic in the model. Furthermore, the full fitted model, which included the variables offspring age as both a linear and a quadratic variable, complexity of the food item, frequency of the food item, the interaction between the linear age variable and the complexity variable, and the random effects, fitted the data better than the null model which only included the random effects (likelihood ratio test: χ^2^ = 19.05, df = 5, p = 0.002), indicating an overall effect of the five variables on the response variable. In terms of the individual effects, the full model revealed a significant negative quadratic effect of the offspring’s age on the food solicitation success as well as a significant interaction between the linear age variable and the complexity variable on the food solicitation success (Table 1: χ^2^_quadratic age_ = 11.42, df_quadratic age_ = 1, p_quadratic age_ = 0.001, χ^2^_linear age: complexity_ = 5.71, df_linear age: complexity_ = 1, p_linear age: complexity_ = 0.02).

**Figure 3:**
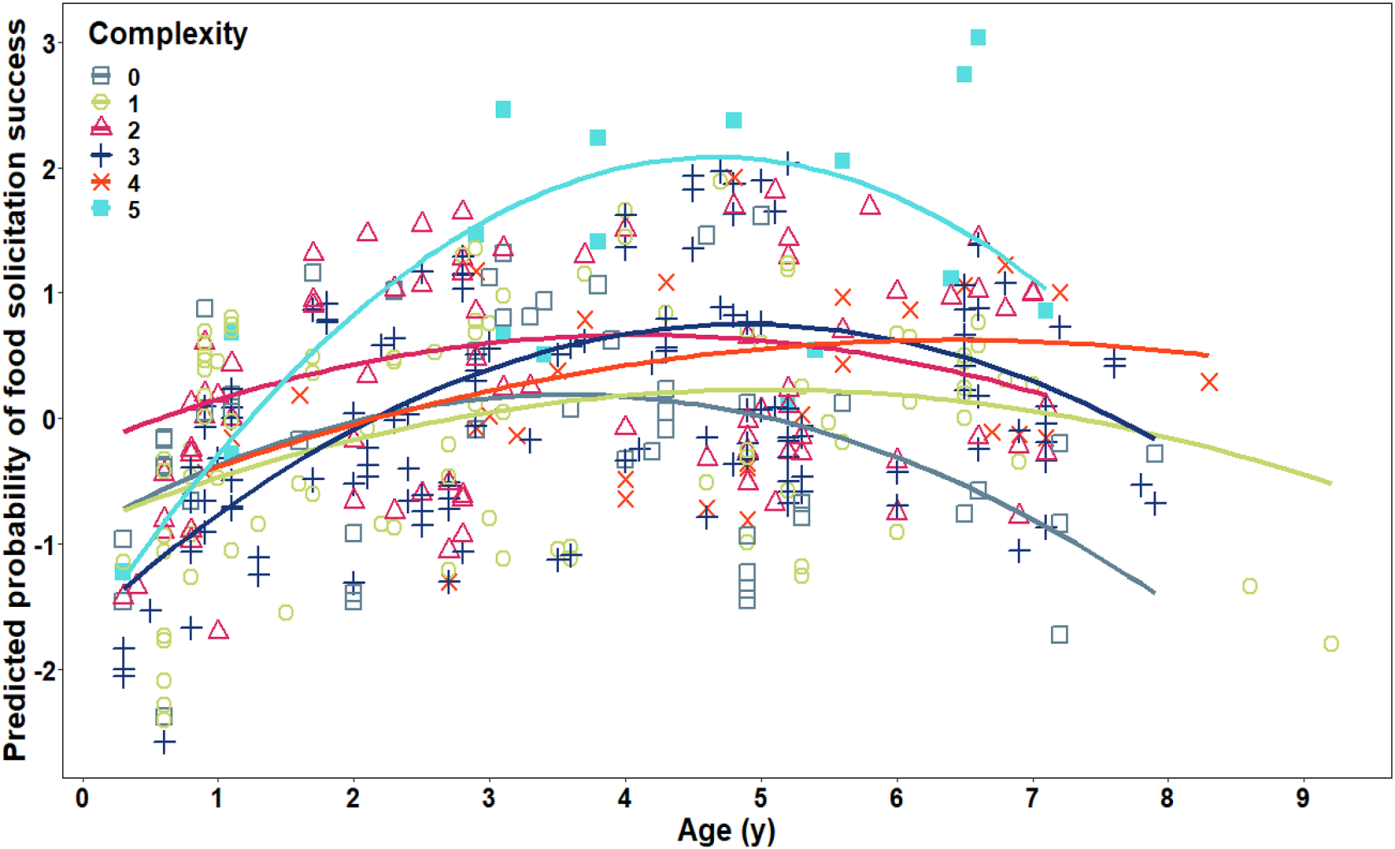
Effects of the age of the offspring and the complexity of the solicited food item on predicted food solicitation success. Complexity was assessed via the number of manual and oral steps it took to process a food item before ingestion and ranged from 0 to 5. The curves show the fitted probability at the 95% level (model 3*, dataset 3*, N = 918 food solicitation events).

Food solicitation success peaked at the age of five and then decreased (Fig. 3). The nature of the interaction effect between age and food solicitation success indicated that with increasing processing complexity of the food item, food solicitation success increases faster until age five, and then decreases more slowly (Fig. 3). Accordingly, the most complex items showed the highest food solicitation success (Fig. 3). The full model indicated no effect of the frequency of the food item on food solicitation success in Table 1.

## Discussion

This study investigated whether (I) immature Sumatran orangutans use food solicitation as a means of acquiring feeding skills and (II) if their mothers facilitate their learning process by adjusting their tolerance levels during food solicitation events. We therefore investigated (I) the effect of the age of the offspring on the overall frequency of food solicitations, as well as the effects of the age of the offspring, complexity of the food item, the interaction between these two variables, and frequency of the food item on the rates of food solicitations (corrected for opportunities to solicit food); and (II) the effects of offspring age, complexity of the food item, the interaction between these two variables, and frequency of the food item in the diet on the mother's tolerance (i.e., the success of the food solicitation event).

### Prediction 1

Our results on food solicitation frequencies and rates indicate that immature Sumatran orangutans solicit food less often as they get older, which is in line with what has been found for Bornean orangutans^10^. Furthermore, we found that the immatures’ food solicitation rates increased with increasing pre-ingestive processing complexity of the food item and that the decrease in solicitation rates over age is slower for complex than for easy to process food items. The data show that throughout their dependency period, immature orangutans solicit complex to process food items from their mothers at a high rate, which is in line with the finding that they reach processing competence for these items around the end of the dependency period only or even later^6^.

However, contrary to results on Bornean orangutans^10^, we did not find an effect of the frequency of the food item on the food solicitation rate. A previous study showed that in Bornean and Sumatran orangutans, rates of observational social learning (i.e., peering) increase with decreasing food item frequency^7^, which suggests that immature orangutans pay more attention to items that they see rarely. However, for the Sumatran orangutans, there appears to be no such effect for food solicitation rates. Notably, most of the rare food items are quite easy to process (i.e., leaves of rare vines). Therefore, it might be that observing their mothers eating these items is enough to learn about these items and no interaction with them is needed. In other words, it might be that in order to learn about rare food items, there is no need to solicit these items.

Nutritional needs of the immatures are a frequently suggested alternative explanation for patterns in food solicitation behaviour across different types of food items and age of the immatures (nutritional hypotheses^32^). The nutritional hypotheses predict that immature primates solicit nutritious food items from their mothers more frequently and successfully because those items provide crucial energy for growth^32^. The findings of several studies on primates^17,73,74^ and non-primates support this prediction^75,76^. Even though we do not have detailed nutritional data available for the food items of the Suaq Balimbing population, it is apparent that many of the complex food items are indeed highly nutritious (e.g., meat of vertebrates, insects and their products, the seeds of complex to process fruits, etc.). Therefore, nutritional needs might be an alternative explanation to the increase in food solicitations and their success with increasing complexity of the food item. Furthermore, complex processing may be more salient, especially if tools are involved, and may thus attract more attention. The decrease in food solicitations with increasing age and competence is also in line with the nutritional hypotheses: as the offspring get better at feeding themselves, they need less nutritional support from their mothers. Furthermore, increased begging for difficult items may be a direct result of those items not being accessible to the offspring. However, one part of the nutritional hypotheses also assumes that food solicitations and their success peak during the period of weaning, because this is when immatures are likely to experience the greatest nutritional needs^32^. Our results did not support this pattern as they showed that by the age of weaning, solicitation frequencies and rates as well as their success have decreased.

In combination, these results on the effect of age on food solicitation frequencies, the effects of age, item complexity, and age interacting with the complexity on food solicitation rates are in line with the informational hypothesis^32^ as they suggest that immature orangutans learn how to handle food items, in particular difficult-to-process items. However, these results are also in line with the nutritional hypotheses in that immature orangutans solicit food because they have to fill their empty stomachs.

### Prediction 2

We found significant effects of the offspring’s age and the complexity of the food item on the mother’s tolerance during food solicitations. Interestingly, the offspring's age had a negative quadratic effect on the mother's tolerance: until the age of 5 with increasing age of the offspring, mothers showed an increasing level of tolerance to food solicitation requests of their offspring, after which tolerance levels decreased again. Furthermore, as predicted, there was a significant interaction between age of the offspring and complexity of the food item. The nature of the interaction effect showed that throughout the dependency period of their offspring, orangutan mothers are more tolerant when their offspring solicit items that need many processing steps compared to less complex food items.

In the first year of life, orangutan immatures rely merely on their mothers' milk and only start ingesting solid food between one and one and a half years of age^3,77^. It is likely that during their first years, immatures cannot digest everything their mothers eat. It would therefore make sense that mothers restrict their food sharing during that time (which could be regulated by a variety of mechanisms). By the age of five, orangutans have started to eat foods of all types but are still increasing their levels of competence in processing these foods^78^. Accordingly, the peak of orangutan mothers' tolerance to their offspring's food solicitation at around the age of 5 years coincides with the period (0–5 years of age^10^) during which immatures solicit food most frequently. High tolerance towards young immatures suggests that the mothers adjust their behaviour to their offspring's low-level of skill, thus supporting their learning. The gradually decreasing tolerance of their mothers during food solicitation events forces immature orangutans to feed on their own and importantly, to practice their emerging feeding skills, as found in other primates^12,79^.

Our results showed that the peak of the maternal tolerance during food solicitations does not coincide with weaning, as would be predicted by the nutritional hypotheses^32^. However, at the age of 5 years immature orangutans likely have high energetic needs because they grow at a fast pace^6,80^ and are only rarely carried by their mothers^3^. Therefore, it may well be that the high maternal tolerance during food solicitations around the age of 5 years also provides nutritional benefits for the offspring and as such supports their physical development, on top of supporting learning as we described above. Once the orangutan offspring grows older than five, the mother's tolerance, and thus her investment in the form of food sharing during food solicitation, gradually decreases. This finding suggests that once the offspring have reached a certain skill level, mothers start to save their energy, preparing themselves for their next offspring^81^.

In general, older offspring have increased motor skills and physical strength and could, therefore, be better at taking food from their mothers. This might be an alternative explanation to the increase in successful food solicitations with increasing age. However, if this were the case, we would see this trend of increasing success to be continued throughout the immature period, which is not what we found. Furthermore, an unpublished study on a subset of the same data used here found that only 11% of all food solicitation events entailed elements of mother–offspring conflicts at Suaq Balimbing (such as agonistic behaviours or crying)^82^. There is no evidence for an increase of conflicts during food solicitation events with increasing age of the offspring^82^. Nevertheless, the physical condition of the offspring might be an important element that regulates food solicitation events in a more indirect way. If the offspring's physical condition is poor in relation to its age, it makes sense for the mother to be more tolerant or to prolong her investment to increase its survival chances. With future data collection it would become possible to test this prediction, given that the current physical condition of the offspring can be assessed reliably (e.g., via body size and growth^83–90^).

In terms of the effect of food complexity on the solicitation success, in a previous study it was shown that immatures reach adult-like food processing skills for complex to process items later during development than for easy to process ones^6^. Furthermore, immature orangutans obtain food processing competence with repeated exposure to mother's manipulating complex foods^6,7^. We found a positive interaction effect of the complexity of the food item with the age of the offspring on food solicitation success. This suggests that throughout their offspring’s development, orangutan mothers are more tolerant when their offspring solicit difficult-to-process items compared to items with fewer processing steps. Based on our data, we cannot make any inferences about the underlying mechanism that regulates this process, i.e., we do not know if (or if so, to what degree) orangutan mothers are aware of the competence levels of their offspring, or the complexity of the food as opposed to being led by innate regulatory mechanisms. A similar question was faced in the case of meerkats' teaching, where researchers experimentally manipulated the offspring's age-cues to show that meerkat adults simply reacted to the presented voice-cue indicating offspring age, and thus ignored the actual skill level of the pups^26,91–93^. In contrast, some primates like cottontop tamarins may have the ability to monitor the knowledge state of immatures and thus to adjust their teaching to, for instance, slow learners^20^. According to Byrne and Rapaport^94^, the main behaviours to indicate awareness of the learner's competence level would include adjusting one's teaching accordingly. In Sumatran orangutans the flexibility in food-sharing behaviours that we found in the present study do not follow a simple linear pattern, as would be expected for a simple age-triggered teaching sequence (e.g., Fig. 3). Further, it is possible to infer the mothers' awareness of their offspring competence using other available proxies. These proxies include some of the cognitive skills known to be implicated in teaching in humans (also in children): monitoring the learner's actions and error recognition^95,96^, theory of mind^95,97^, and adaptability to respond dynamically to the learner's progress^98,99^. Which of these elements of teaching cognition are present in orangutans? Adult orangutans show action recognition^100^ and theory of mind^101^ (but see^102^). In our long-term data we have noted instances when orangutan mothers adapt their behaviour to their offspring's knowledge state, such as when preventing the immature from eating faeces or poisonous food items (Schuppli, personal communication). Collecting more data across a larger number of individuals will allow to investigate the flexibility of this mechanism (i.e., in terms of reactions to specific needs of individual offspring based on their specific skill levels or the point of processing at which the mothers share the food item) which may shed light on the cognitive mechanisms at work.

Being aware of the competence levels of the observer is not a prerequisite of functional teaching^103^. To what extent do our results as such support the presence of teaching in wild orangutans? Of the three criteria listed by Caro and Hauser^37^, supporting the first one, i.e., that the actors alter their behaviour in the presence of a naive observer, we found that the tolerance of the mothers decreased with increasing age and thus competence level of the offspring. Furthermore, in line with the second criterion, i.e., that the behaviour needs to come without immediate benefits or at a cost to the actor, giving food away has no immediate benefit for the mother and likely leads to opportunity costs for her. However, our data do not allow for testing for the third criterion, namely that food solicitations lead an increase in learning speed in the offspring. In short, we found evidence for two criteria of functional teaching but cannot test the third.

Less than one percent of the food solicitation events in our study were initiated by the mother. This means that even though orangutan mothers’ behaviour likely facilitates their offspring skill acquisition, this happens in a reactive, rather than proactive way. Therefore, in orangutans, learning through food solicitations is dependent on the initiative of offspring. Our results also contrast with results obtained from chimpanzees which showed that mothers actively facilitate the acquisition of complex feeding skills (i.e., tool assisted termite-fishing or nut-cracking) in immatures by adjusting their tolerance levels during food solicitations and by sharing foraging tools^41,42,70,104^. In humans, teaching plays a major role during skill acquisition^105,106^ even though it is debated how common proactive teaching is in everyday skill acquisition outside the modern education setting^107–109^. However, apart from teaching, there are other, more passive processes at work in humans which resemble the pattern we found for the orangutans in our study: through social referencing, children actively seek out information from role models to appraise situations while there is no active involvement of the role model in the learning process as such^110,111^. Our results on the orangutan mothers’ reactions during food solicitations suggest that a similar mechanism may be at work in orangutan feeding skill acquisition, but we cannot draw any inferences about the emotional state of the mothers^112^.

Therefore, even though our results suggest that wild orangutan mothers guide the learning process of their infants through behavioural adjustments, they are less proactive than two of their closest relatives, chimpanzees, and humans. In general, mothers are expected to actively invest in helping their offspring’s skill acquisition if the benefits of doing so outweigh the costs^103^. Orangutans face higher energetic constraints than the other apes, due to their higher degree of arboreality and the comparably low productivity of Asian rainforests^113,114^. These energetic constraints may make orangutans more susceptible to costs resulting from active changes in behaviour or missed feeding opportunities. The cost benefit ratio depends also on the ease with which young can learn these skills without active involvement of the mother^103^. It may be that the feeding skills immature orangutans need to learn are less complex than those of humans and chimpanzees and can thus be acquired through cues obtained from reactive behavioural adjustments only. Furthermore, contrary to humans and chimpanzees, orangutan infants may have a larger focus on their mothers because they spend most of time being in close proximity^2,3^, which may make it easier for them to pick up social cues of less active role models. Strikingly, the small amount of active sharing among wild orangutans is not in line with what has been found for captive Sumatran orangutans, among whom there is frequent active food sharing even in non-mother–offspring dyads^115^. These results suggest that orangutans are capable of active sharing but do not do so in their natural habitat.

In terms of the relationship between the frequency of the food items and the mother's tolerance, previous research found that immature orangutans peer more frequently with decreasing frequency of the food item when correcting for the opportunities they receive to peer at different food items^7^. This finding suggests that the fewer opportunities immatures have to learn about specific food items, the more they make use of these opportunities when they do occur. Hence, if orangutan mothers were to facilitate the learning process of their offspring, they should be more tolerant for less frequent items their immatures solicit. However, against our prediction we found no effect of the frequency of the food item on the mother's tolerance. A possible explanation for this result is that the mothers are more reluctant to share uncommon food items because those uncommon food items may provide the mothers with micro and macronutrients that are important for milk production^77^. Another possibility is that the immatures may not be able to digest these uncommon food items^116^. Furthermore, especially when it comes to mothers trying to add new items to their diets (i.e., sampling new potential food items), it is safer not to let the immatures eat them because these items could potentially be harmful. This negative result also reflects our result on the food solicitation rates which showed that immatures did not differentiate between rare and common food items in their food solicitation behaviour (see above). Together, the absence of any effects of food item frequency on food solicitation rates and food solicitation success results imply that soliciting food is not used to learn or facilitate learning specifically about rare items.

## Conclusions

Overall, our results showed that immature Sumatran orangutans solicit food during the time they learn how to process food and that they reduce solicitation frequencies as they grow older and more skilled. Also, immatures solicit food items which require multiple processing steps more frequently than easy to process ones. These results support both, the informational and nutritional hypotheses. Furthermore, their mothers reactively adjust their tolerance level to the food solicitation behaviour depending on the complexity of the food item and their offspring’s age, in that they are more tolerant when more difficult items are being solicited and most tolerant during the time their offspring learn their feeding skills. These patterns likely facilitate the offspring’s foraging skill acquisition. In conclusion, immature Sumatran orangutans learn about their diets via food solicitation, and their mothers play a more active role in the feeding skill acquisition of their immature offspring than previously assumed.

## Supplementary Information

Supplementary Information.

## Data Availability

The datasets generated during and/or analysed in the current study are available in the Harvard Dataverse repository, https://dataverse.harvard.edu/dataset.xhtml?persistentId=doi:10.7910/DVN/KHEUES.
