# Supplementary material for: Orangutan mothers adjust their behaviour during food solicitations in a way that likely facilitates feeding skill acquisition in their offspring
Source: Sci Rep. 2021 Dec 8;11:23679. doi: 10.1038/s41598-021-02901-z (PMC8655057; doi:10.1038/s41598-021-02901-z)
Supplement: Supplementary file 1 — Supplementary Information. [file 41598_2021_2901_MOESM1_ESM.pdf]

Orangutan mothers adjust their behaviour during food solicitations in a way that likely facilitates feeding skill acquisition in their offspring

Mulati Mikeliban, Belinda Kunz, Tri Rahmaeti, Natalie Uomini, Caroline Schuppli *

Supplementary Table 1. General information on all 27 offspring of this study. Columns from left to right: name, age ranges (in years) from their first available to the last available food solicitation event during the study period (rounded to half-year intervals), name of the mother, sex, how many observation hours we had available across the study period, participation in the different datasets (1-3; the exclusion criteria for each dataset are outlined in the methods section), and the total number of food solicitation events by the individual across all the datasets. Notably, because the exact date of birth of the free-ranging orangutans is often unknown, their birth dates were estimated by experienced researchers and based on their developmental characteristics when they were first found.

Focal	Age ranges	Mother	Sex	Follow hours	Dataset(s)	Number of food solicitations
Albin	4.5	Alice	male	11.4	1,2,3	2
Amor	0.5, 2.5-4.0	Alice	male	45.6	1,2,3	8
Cheech	7.0	Chick	male	1.47	2	0
Chindy	5.0-6.0, 8.0,14.5	Cissy	female	150.9	1,2,3	34
Chuck	1.0	Chick	male	3.6	3	1
Cinnamon	2.0-3.0, 4.5-5.5, 6.5, 7.5	Cissy	female	528.6	1,2,3	156
Dalia	2.0	Dodi	female	33.5	1,2,3	6
Diddy	5.0, 8.0	Dodi	male	22.13	2,3	6
Eden	0-0.5, 2.0-4.0, 4.5-5.0	Ellie	female	794.6	1,2,3	228
Ellie	9.0, 10.0, 11.5-12.0	Friska	female	167.6	1,2,3	3
Frankie	1.0, 1.5-2.0, 4.5-6.0, 6.5-7.0	Friska	male	686.4	1,2,3	328
Fredy	2.5-3.0, 3.5, 4.0, 6.0, 9.0, 10.0	Friska	male	484.5	1,2,3	156
Ian	-	Intai	male	6.77	3	1
Leon	0.5	Lisa	male	3.83	2	0
Lilly	6.5-7.0, 8.0, 10.0, 13.0, 14.0	Lisa	female	257.5	1,2,3	66
Lois	0.5, 3.0-4.0, 6.5-9.5	Lisa	male	765.0	1,2,3	177

Luther	1.5, 3.0	Lilly	male	58.5	1,2,3	23
Pepito	4.5, 6.5	Piniata	male	34.6	1,2,3	16
Rendang	1.0	Raffi	male	118.2	1,2,3	26
Ronaldo	1.5-2.0	Raffi	male	116.0	1,2,3	85
Sazu	6.5	Sarabi	male	10.8	1	0
Shera	13.0	Chick	female	80.5	1	0
Simba	0.5-1.0	Sarabi	male	129.6	1,2,3	62
Tina	10.0-11.0, 13.0	Raffi	female	105.4	1	0
Tornado	3.0, 4.0	Tiara	male	22.4	1,2,3	4
Trident	10.5	-	female	33.6	1	0
Yulia	10.0, 11.0-11.5	-	female	144.0	1,3	2

Supplementary Table 2. Age variable being linear vs being quadratic in the models.

Statistical model	npar	logLik	deviance	χ^2	*df*	P
**Model 1**						
Age variable is linear	6	-1.588	3.1764	-	-	-
Age variable is linear and quadratic	7	0.8922	-1.7845	4.96	1	**0.026**
**Model 2**						
Age variable is linear	6	-1735.0	3470.1			
Age variable is linear and quadratic	7	-1734.6	3469.1	0.92	1	0.337
**Model 3**						
Age variable is linear	5	-577.41	1154.8	-	-	-
Age variable is linear and quadratic	6	-572.95	1145.9	8.92	1	**0.003**

* There is the age variable (linear / linear & quadratic) as the only fixed effect, and the random effects including the offspring and the observer (i.e., the observer and offspring intercepts and the slope of age within the offspring intercept), in each of the models.

Supplementary Table 3. With and without sex variable in the models.

Statistical model	npar	logLik	deviance	χ^2	*df*	P
**Model 1**						
Reduced model (without sex variable)	7	0.8923	-1.7845	-	-	-
Full model (with sex variable)	8	1.2376	-2.4751	0.69	1	0.41
**Model 2**						
Reduced model (without sex variable)	12	-1638.0	3276.1	-	-	-
Full model (with sex variable)	13	-1637.9	3275.8	0.24	1	0.62
**Model 3**						
Reduced model (without sex variable)	12	-555.77	1111.5	-	-	-
Full model (with sex variable)	13	-555.74	1111.5	0.05	1	0.84

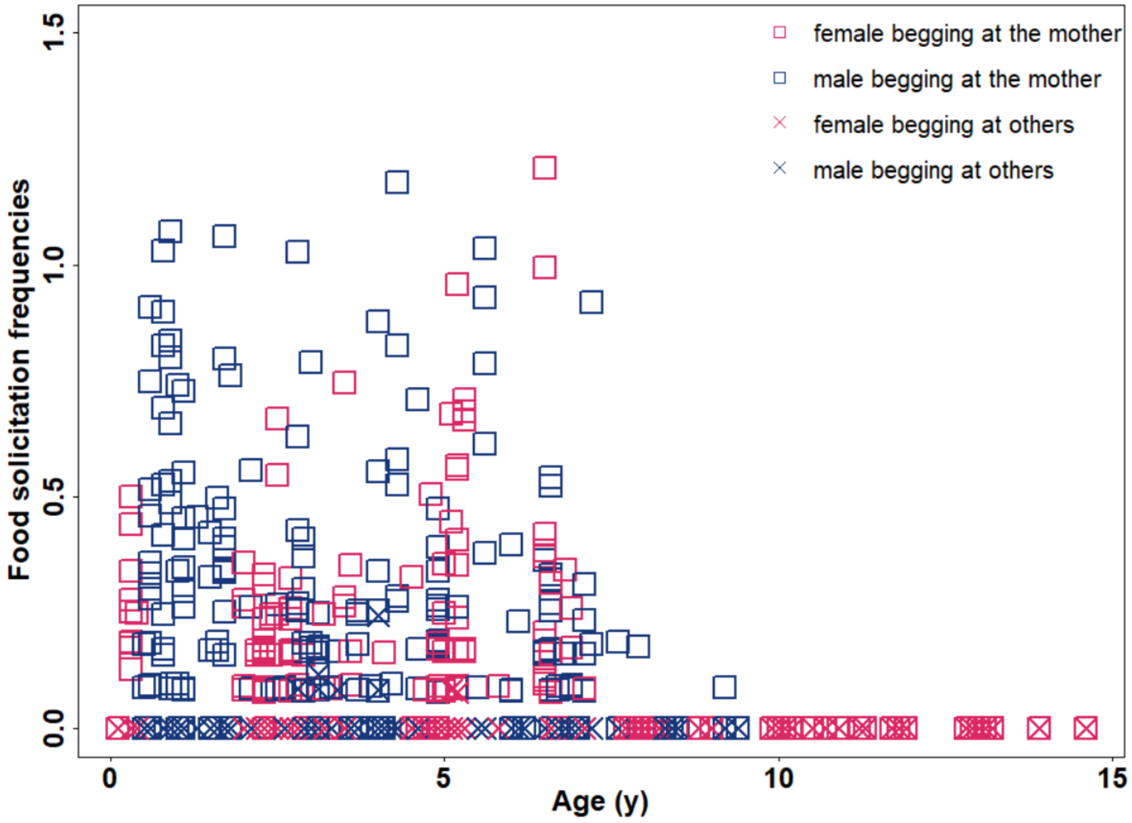

Supplementary Fig. 1. Raw data inspection of food solicitation frequencies (events per hour, calculated per day) over age and sex of the offspring (*dataset 1*, N = 416 food solicitation frequencies).

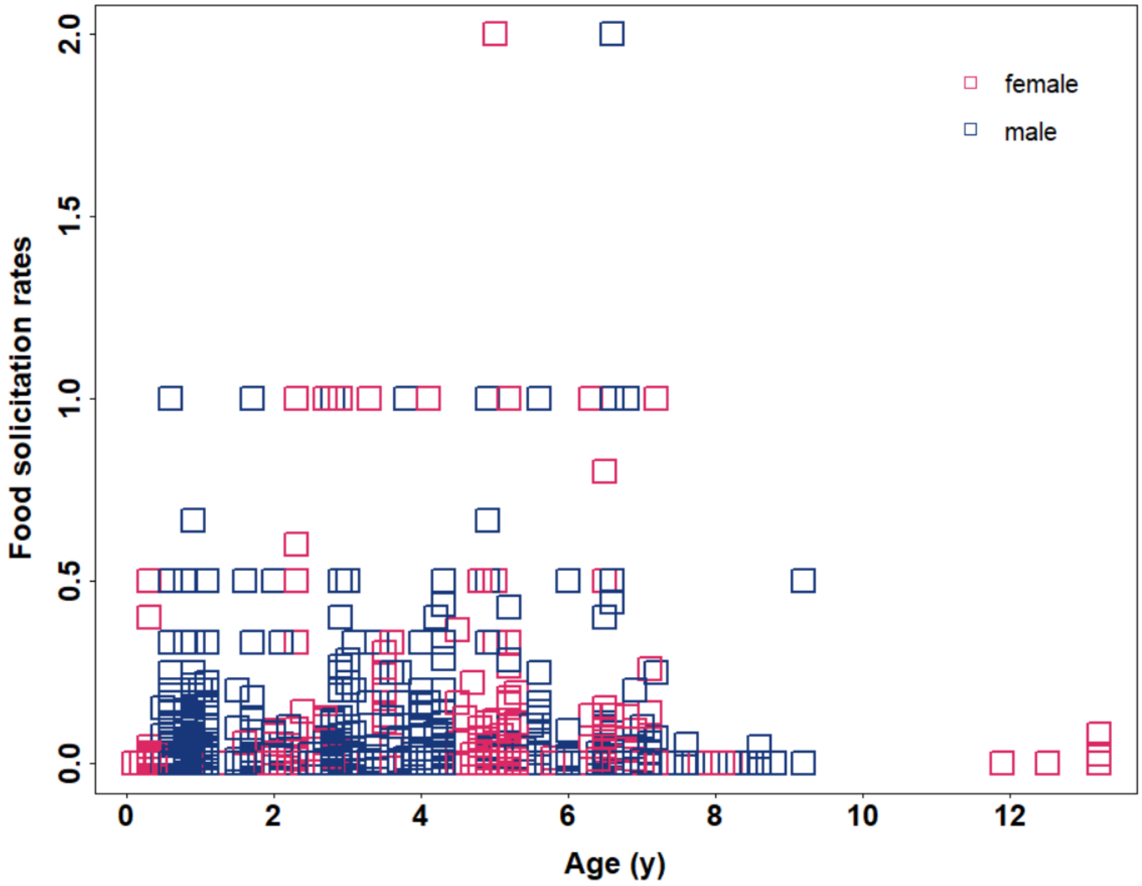

Supplementary Fig. 2. Raw data inspection of food solicitation rates (events per food solicitation opportunity, i.e., per 2-minute scan the mother was feeding on the same item, calculated per day, log-transformed) over age and sex of the offspring (*dataset 2*, N = 3671 food solicitations rates).

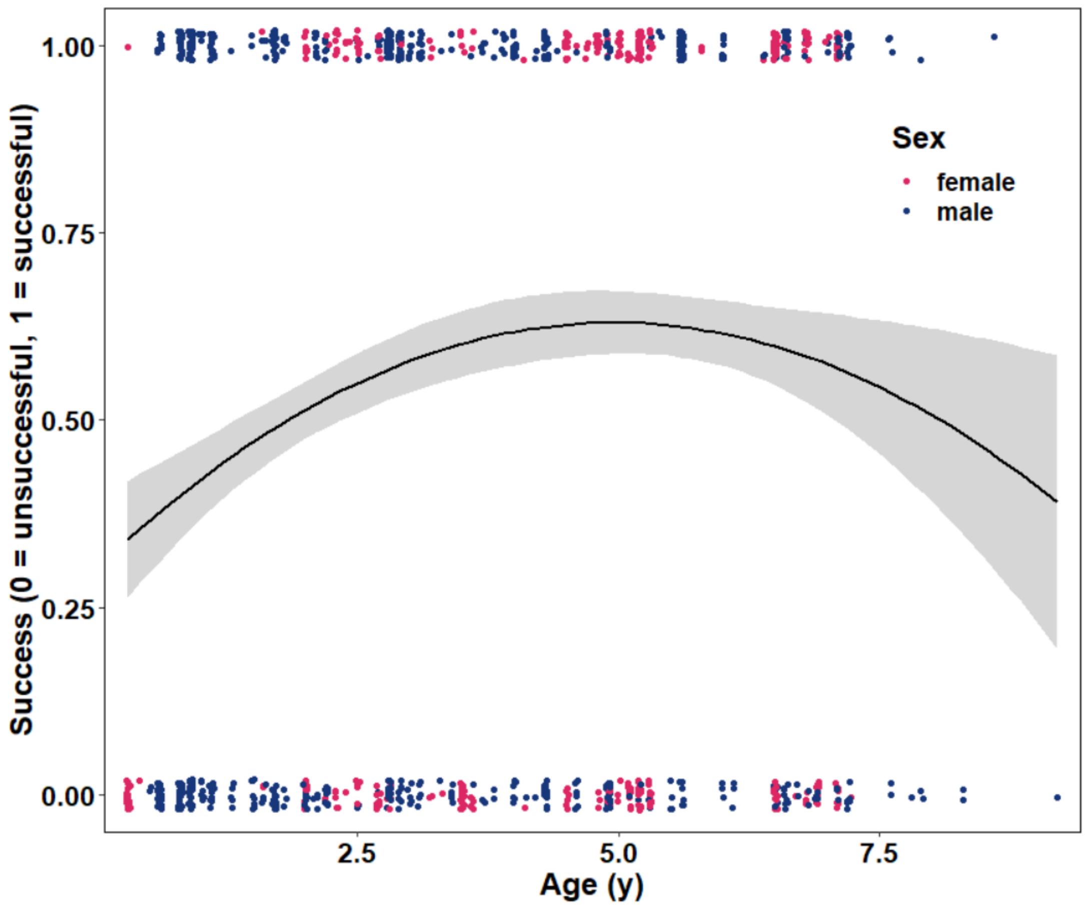

Supplementary Fig. 3. Raw data inspection of food solicitation success over age and sex of the offspring. The dots represent our raw data (*dataset 3*, N = 1379 food solicitation events). The curve shows the fitted probability at the 95% level.
